# The Role of Polyamine-Dependent Facilitation of Calcium Permeable AMPARs in Short-Term Synaptic Enhancement

**DOI:** 10.3389/fncel.2018.00345

**Published:** 2018-10-10

**Authors:** Andrei Rozov, Yulia Zakharova, Alina Vazetdinova, Fliza Valiullina-Rakhmatullina

**Affiliations:** ^1^Laboratory of Neurobiology, Institute of Fundamental Medicine and Biology, Kazan Federal University, Kazan, Russia; ^2^Department of Physiology and Pathophysiology, University of Heidelberg, Heidelberg, Germany

**Keywords:** calcium permeable AMPA, GluA2-lacking AMPA, synapse, short-term plasticity, homeostatic plasticity, neurons, interneurons, polyamine

## Abstract

Depending on subunit composition AMPA receptor channels can be subdivided into two groups: GluA2-containing calcium impermeable AMPARs, and GluA2-lacking calcium permeable, AMPARs. These two groups differ in a number of biophysical properties and, most likely, in their functional role at glutamatergic synapses. GluA2-lacking channels have received a lot of attention over the last two decades mainly due to high calcium permeability, which was suggested to play a significant role in the induction of long-term synaptic plasticity in healthy tissue and neuronal death under neuropathological conditions. However, calcium permeable AMPARs possess another property that can contribute substantially to frequency dependent dynamics of synaptic efficacy. In the closed state calcium permeable AMPARs are blocked by endogenous polyamines, however, repetitive activation leads to progressive relief from the block and to the facilitation of ion flux through these channels. Polyamine-dependent facilitation of AMPARs can contribute to short-term plasticity at synapses that have high initial release probability and express calcium permeable AMPARs. During synaptic transmission activity-dependent relief from polyamine block of postsynaptic calcium-permeable AMPARs either counteracts presynaptic short-term depression in a frequency-dependent manner or, under specific stimulation conditions, induces facilitation of a synaptic response. Taking into account the fact that expression of calcium permeable AMPARs is developmentally regulated, depends on network activity and increases in diseased brain states, polyamine-dependent facilitation of calcium permeable AMPARs is an important, entirely postsynaptic mechanism of synaptic gain regulation.

## Introduction

The functional properties of AMPA receptor (AMPAR) channels are, to a large extent, determined by subunit composition. Single channel conductance and calcium permeability are conferred by the presence or absence of the GluA2 subunit. In contrast to the other AMPAR subunits (GluA1, GluA3, and GluA4), the vast majority (>99%) of GluA2 mRNA undergoes post-translational editing, which converts a codon for glutamine (Q), present in the GluA2 gene, to a codon for arginine (R) at position 607 in the pore forming **M2** segment (Sommer et al., 1991) This alteration of charge in the channel pore, leads to a loss of calcium permeability, strongly reduces the single-channel conductance of the receptor and prevents the channel from being blocked by intracellular polyamines (Verdoorn et al., 1991). Thus, calcium permeable AMPARs (CP-AMPARs) assembled from GluA1, GluA3 and GluA4, show higher single-channel conductance and have an inwardly rectifying current-voltage relationship resulting from voltage-dependent block by intracellular polyamines. Channels containing edited GluA2 are calcium impermeable AMPARs (CI-AMPARs), which have a lower single-channel conductance and exhibit a linear voltage–current relationship (Hestrin, 1993). Over the last two decades, a great deal of attention has been paid to the functional role of the calcium permeability of CP-AMPARs (Pellegrini-Giampietro et al., 1992; Friedman et al., 1994; Gorter et al., 1997; Grooms et al., 2000; Opitz et al., 2000; Huang et al., 2002; Liu and Zukin, 2007; Lu et al., 2007; Guire et al., 2008; Wang and Gao, 2010; Rozov et al., 2012; Sanderson et al., 2016). However, this property of CP-AMPARs comes together with the voltage-dependent block by intracellular polyamines and the ability of CP-AMPARs to be relieved from this block during repetitive high frequency activation. The latter of which results in facilitation of CP-AMPAR mediated currents (Rozov et al., 1998; Rozov and Burnashev, 1999). In this mini-review we discuss the mechanism of polyamine-dependent facilitation of CP-AMPARs and its possible contribution to short-term plasticity at glutamatergic synapses.

## Contribution of Cp-Ampars to Frequency Dependent Facilitation

### Mechanism of Polyamine-Dependent Facilitation of CP-AMPARs

Under normal physiological conditions the calcium permeability of synaptic CP-AMPARs does not contribute much to postsynaptic calcium dynamics (Bollmann et al., 1998), due to the rapid inactivation of AMPAR-mediated responses and the relatively small fractional Ca^2+^ current (P_f_) through these channels. Even in recombinant systems, where the Ca^2+^ permeability of all expressed AMPAR channels can be ensured by transfection of vectors encoding the specific subunit, the P_f_ of homomeric GluA1 CP-AMPARs is about 3% of the total current. For comparison, this value measured for GluN1A-GuluN2 NMDAR channels is three fold higher and exceeds 10% (Burnashev et al., 1995). However, another feature of CP-AMPARs that comes together with high Ca^2+^ permeability, is their sensitivity to intracellular polyamines allows AMPAR-mediated currents to increase in an activity dependent manner.

**FIGURE 1:**
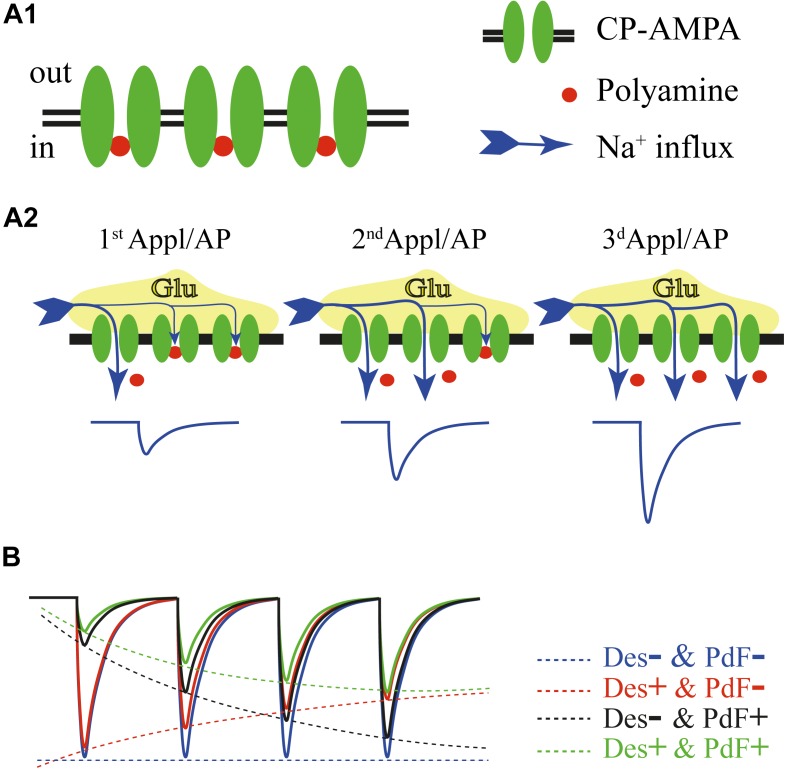
**(A)** Schematic drawing of events underlying polyamine dependent facilitation of CP-AMPARs. In the closed state most of the channels are blocked by intracellular polyamines **(A1)**. Repetitive activation of CP-AMPARs at negative potentials either by a train of brief glutamate applications or by mediator released in response to the train of action potentials, leads to progressive relief from the block and enhancement of AMPAR-mediated current amplitudes **(A2)**. **(B)** Factors determining amplitudes of the currents through CP-AMPARs during high frequency activation. The blue trace shows a hypothetical response to a train of glutamate applications without contribution of channel desensitization and polyamine dependent facilitation. Experimentally this can be achieved in the presence of cyclothiazide and after washout of intracellular polyamines. Contribution of polyamine dependent facilitation (no channel desensitization) is depicted by the black trace. Note the strong impact of the polyamine block on the first response amplitude. The red trace shows gradual reduction of AMPAR-mediated currents due to channel desensitization in the absence of polyamine dependent facilitation. The green trace illustrates the product of interplay between channel desensitization and polyamine dependent facilitation of CP-AMPARs. The final amplitude dynamics in every given case will depend on: desensitization time constants, polyamine concentration and stimulation frequency.

All AMPAR channels composed of GluA1, GluA3, or/and GluA4 subunits can be blocked by endogenous polyamines such as spermidine, and spermine. Blockade of CP-AMPARs by intracellular polyamines generates inward rectification of the current-voltage (I–V) relation. However, as membrane potential becomes sufficiently positive, polyamines can permeate the AMPAR channel so that ion flux is restored resulting in a so called doubly rectifying I–V relation (Bowie and Mayer, 1995; Burnashev et al., 1995). For a while, it was thought that polyamines act only as an open channel blocker, being more powerful at positive potentials when both the transmembrane voltage and the concentration gradients synergistically push them into the inner vestibule of the channel. Since polyamines, depending on their length, can carry up to four positive charges, the presence of these molecules in the path of ion flow greatly reduces conductance of the channel. The same line of logic was applied to explain the “weaker” polyamine block at negative potentials – a reduction of electric driving force for polyamines leads to a decrease in the probability of entering the channel in the open state (Bowie and Mayer, 1995). However, it was later shown that polyamines can enter the inner vestibule of CP-AMPARs in the closed state, and the opening of the channel leads to either relief from the block or complete obstruction of the ion flow, depending on membrane potential (Rozov et al., 1998). At positive potentials polyamines are forced to permeate through the channel according to the transmembrane voltage and the concentration gradients. However, taking into account the size of these organic ions, a substantial electric driving force is needed to push polyamines through the narrow constriction of AMPARs. Even when the intracellular concentration of free polyamines is in the range of a few micromoles, a measureable outward CP-AMPAR mediated current can be detected at potentials higher than 20 mV (Donevan and Rogawski, 1995; Kamboj et al., 1995; Rozov et al., 1998). At negative potentials, opening of the CP-APMARs leads to relief from the block, since due to the transmembrane potential difference, polyamines have to leave the channel pore back to the cytosol (**Figure 1A**). The rate of relief strongly depends on holding potential, being faster at more negative potentials (Rozov et al., 1998). Given that release from the block is electrically driven, and the speed of re-block depends on polyamine diffusional constants, repetitive activation of the same population of CP-AMPARs results in progressive liberation of these channels from polyamine suppression and can therefore cause facilitation of AMPAR-mediated currents. Indeed, in outside-out patches expressing homomeric CP-AMPAR channels composed of GluA1, GluA4, or unedited GluA2 subunits, trains of 1 ms glutamate applications induced inward currents which showed robust facilitation at application frequencies of 2 Hz or greater. The crucial role of the polyamine block in this phenomenon was further proven by experiments where washout of polyamines lead to the loss of facilitation. Moreover, the I–V relation of the facilitated responses was almost linear, even in the presence of polyamines, pointing to activity dependent relief from the block as the main mechanism of CP-AMPAR facilitation (Rozov et al., 1998).

The apparent degree of current facilitation also dependents on the AMPARs desensitization properties. For instance, GluA1-containing AMPARs, which show the slowest recovery from desensitization of all ‘flip’ form subunits, showed strong depression at application frequencies higher than 10 Hz. Still, in the presence of cyclothiazide, a positive allosteric modulator that eliminates channel desensitization, currents through GluA1-containing AMPARs were facilitating in response to 33 Hz application trains. It is important to state that desensitization and polyamine-dependent facilitation of CP-AMPARs are two independent processes (**Figure 1B**). Even at high application frequencies, when the desensitization impact on response amplitudes is strong, the presence of intracellular polyamines selectively reduces the amplitude of the first CP-AMPAR mediated response and consequently diminishes the depth of the relative depression (Rozov et al., 1998).

### The Role of Polyamine-Dependent Facilitation of CP-AMPARs in Short-Term Synaptic Plasticity

Polyamine-dependent facilitation can operate as an entirely postsynaptic mechanism of short-term synaptic enhancement at synapses expressing CP-AMPARs. The main requirement for significant contribution of this phenomenon to short-term plasticity is high release probability, since the same population of synaptic CP-AMPARs should be exposed to glutamate at every cycle of the presynaptic activity. Excitatory synapses onto many interneurons express CP-AMPARs and are characterized by initial high release probability. One of the most studied types of principal cell to interneuron synapses, the connection from cortical pyramidal neurons to parvalbumin positive fast spiking (FS) interneurons, provides a good model to demonstrate the involvement of polyamine-dependent facilitation in synaptic plasticity for the two following reasons. Firstly, most of the described synapses of this type have a release probability that is close to one (Reyes et al., 1998; Angulo et al., 1999; Rozov et al., 2001; Watanabe et al., 2005; Hull et al., 2009; Voinova et al., 2015). Secondly, FS cells express a high level of CP-AMPARs postsynaptically, as is the case in the vast majority of cortical interneurons (Geiger et al., 1995; Koh et al., 1995; Toth and McBain, 1998; Rozov and Burnashev, 1999). Indeed, it has been shown that in connected layer 2/3 pyramidal to FS cell pairs, trains of presynaptic action potentials at stimulation frequencies of 10 Hz or higher trigger postsynaptic responses exhibiting prominent paired-pulse depression. However, reduction of the second EPSC amplitude relative to the first one was significantly stronger after washout of polyamines from the postsynaptic interneurons. In the presence of intracellular spermine the I–V relation of the first EPSC was sigmoidal, while the negative limb of synaptic I–V of the second EPSC was linear, suggesting significant reduction of polyamine block. Moreover, when vesicle depletion was reduced by lowering extracellular Ca^2+^ concentration (from 2 to 1.5 mM) 10 Hz stimulation of excitatory inputs to layer 2/3 FS interneurons evoked EPSPs that showed paired-pulse facilitation in the cells patched with spermine-containing intracellular solution. However, in the simultaneously recorded FS neurons dialyzed with polyamine-free pipette solution, the same train of presynaptic action potentials triggered depressing postsynaptic responses (Rozov and Burnashev, 1999).

Toth et al. (2000) provided further evidence that polyamine-dependent facilitation of CP-AMPARs can serve as an entirely postsynaptic mechanism of short-term plasticity. They found that at CP-AMPA expressing synapses formed by mossy fibers onto stratum radiatum interneurons, depolarization of the postsynaptic cell from -80 to -20 mV leads to facilitation of the EPSCs evoked by 20 Hz afferent stimulation trains. Indeed, as was previously shown on recombinant CP-AMPARs, polyamine-dependent facilitation measured as paired-pulse ratio (PPR) is more pronounced at less negative potentials and requires longer application trains to reach the steady-state level, since the rate of relief is voltage dependent (Rozov et al., 1998). Thus, polyamine-dependent facilitation can maintain the amplitude of the postsynaptic responses counteracting presynaptic depression at resting membrane potentials, or even switch the modality of short-term plasticity to facilitation upon depolarization of the postsynaptic neuron.

Involvement of CP-AMPARs in synaptic transmission (Rozov et al., 2012) and short-term plasticity has also been shown for connections from hippocampal CA3 to CA1 pyramidal neurons (Mattison et al., 2014). Mattison and colleagues showed selective expression of CP-AMPARs in the dendritic spines located on apical, but not basal, dendrites of CA1 pyramidal neurons using the following approaches: (i) Bath application of spermine causes use-dependent inhibition of excitatory responses evoked by stimulation in the stratum radiatum, but not stratum oriens; (ii) Photostimulation (microphotolysis of caged glutamate) of single dendritic spines induces AMPAR-mediated calcium influx only at apical spines; (iii) Bath application of glutamate lead to AMPAR-mediated Co^2+^ loading only into apical dendrites of CA1 pyramidal cells. Moreover, they found that responses triggered by photostimulation of single apical spines displayed prominent paired-pulse facilitation. The amplitudes of induced EPSCs in apical dendrites were reduced, but PPR was increased, by spermine. Finally, suppression of the recycling of GluA2-containing AMPARs caused reduced apical spine responses and increased PPR. All of these observations strongly suggest both a significant impact of CP-AMPARs on synaptic efficacy and an important contribution of polyamine-dependent facilitation in short-term plasticity at stratum radiatum CA3 to CA1 pyramidal cell synapses (Mattison et al., 2014).

### Polyamine-Dependent Facilitation of CP-AMPARs During Development

Expression of CP-AMPARs can be developmentally regulated. For instance, about 30% of FS interneurons in the rat prefrontal cortex express mostly calcium impermeable AMPARs during the first five postnatal weeks and in adulthood (11 weeks and older), while between these two ages (5 weeks to 10 weeks) 90% of FS cells of this cortical region have synaptic AMPARs that are highly sensitive to polyamines (Wang and Gao, 2010). Interestingly, in those interneurons that express predominantly CP-AMPARs, endogenous and exogenously loaded polyamines trigger synaptic facilitation of AMPA-mediated EPSCs in response to high frequency presynaptic stimulation. Contrarily, in the depressing synapses onto interneurons expressing CI-AMPARs the PPR did not depend on spermine concentration. The contribution of the developmental enhancement of CP-AMPAR expression and increase of polyamine-dependent facilitation to synaptic plasticity have also been demonstrated for local excitatory inputs to layer 2/3 FS cells in the mouse visual cortex (Lu et al., 2014).

Similarly to the synapses onto FS interneurons in the prefrontal cortex, at some synapses between excitatory neurons CP-AMPAR expression is developmentally regulated being higher in younger animals (Shin et al., 2005, 2007; Ho et al., 2007). At hippocampal mossy fiber to CA3 pyramidal cell connections CP-APMARs contribute significantly to the amplitude of the postsynaptic response in mice younger than P17, while in older animals the vast majority of postsynaptic channels are GluA2-containing CI-AMPARs. In slices from P10–P16 mice, evoked EPSCs have I–V relationships with clear inward rectification and application of the CP-AMPAR specific antagonist philanthotoxin-433 causes a strong reduction in their amplitudes. During this developmental stage the PPR of EPSCs evoked by 20 Hz trains of mossy fiber stimulation were significantly larger when postsynaptic cells were held at **-**60 mV compared to that recorded at +40 mV (Ho et al., 2007). The latter observation is in good agreement with activity dependent relief from the polyamine blocks of postsynaptic CP-AMPARs. Similar transient expression of CP-AMPARs by principal neurons early in development has been shown for neocortical layer 5 pyramidal neurons (Shin et al., 2005, 2007). In P12-P14 rats, evoked AMPA-mediated EPSCs were sensitive to intracellular polyamines: (i) I–V relationships showed robust inward rectification; (ii) washout of polyamines lead to increase of EPSC amplitudes and linearization of I–V. During the same developmental period evoked EPSCs at these synapses are characterized by profound paired-pulse facilitation that is crucially dependent on the presence of cytosolic polyamines. In older rats (>P16) synaptic AMPARs had a linear I–V relation and the amplitude of responses as well as PPRs were not affected by either polyamine washing or washout (Shin et al., 2005, 2007). Increased expression of CP-AMPARs and polyamine-dependent facilitation of AMPAR-mediated EPSCs at early developmental stages have also been shown for layer 2/3 pyramidal cells and layer 4 stellate cells (Brill and Huguenard, 2008). Interestingly, synthesis of polyamines in pyramidal neurons is developmentally regulated. In young animals, the levels of spermine and its key metabolic enzyme ornithine decarboxylase are increased, and high expression of polyamines coincides in time with expression of CP-AMPARs (Shin et al., 2005), emphasizing the critical role of polyamine-dependent facilitation in brain development.

## Factors that can Influence the Contribution of Polyamine Dependent Facilitation of Cp-Ampars to Short-Term Plasticity

### Regulation of CP-AMPAR and Polyamines Expression Under Physiological and Pathophysiological Conditions

Obviously polyamine-dependent facilitation can operate only in those synapses where polyamines are expressed and CP-AMPARs significantly contribute to the postsynaptic response.

In a study published by Plant et al. (2006) it has been suggested that at Schaffer collateral to CA1 cell synapses, CP-AMPARs can be temporally recruited during LTP induction. In contrast, no involvement of CP-AMPARs has been seen in hippocampal CA1 LTP in other studies (Adesnik and Nicoll, 2007; Gray et al., 2007). However, there is a growing body of evidence in support of the first idea that functional expression of synaptic CP-AMPARs can increase temporarily in response to some LTP-inducing stimuli and/or bust of activity (Lu et al., 2007; Guire et al., 2008; Yang et al., 2010; Rozov et al., 2012; Sanderson et al., 2016). In all reported cases after LTP induction potentiated EPSCs showed either increased sensitivity to endogenous polyamines or could be partially blocked by selective CP-AMPAR antagonists. Interestingly, silencing of network activity also leads to the enhancement of CP-AMPARs representation at the number of synapses. This form of homeostatic plasticity has been reported in both *in vitro* and *in vivo* models (Wong-Riley and Jacobs, 2002; Bai and Wong-Riley, 2003; Thiagarajan et al., 2005; Goel et al., 2011; Soares et al., 2013; Kim and Ziff, 2014). In addition, CP-AMPARs are recruited to synapses in the amygdala during fear learning (Clem and Huganir, 2010) and in the nucleus accumbens and ventral tegmentum in models of drug addiction (Bellone et al., 2011; McCutcheon et al., 2011) CP-AMPARs have a crucial role not only in synaptic plasticity, but also in the excitotoxicity associated with several neurological disorders. In CA1 pyramidal cells ischemic insults leads to downregulation of GluA2 expression increasing the contribution of CP-AMPARs at theses synapses (Pellegrini-Giampietro et al., 1992; Gorter et al., 1997; Opitz et al., 2000). In hippocampal pyramidal neurons GluA2 expression can be markedly suppressed by seizures which also shifts synaptic AMPAR identity toward CP-AMPARs (Friedman et al., 1994; Grooms et al., 2000; Huang et al., 2002; Liu and Zukin, 2007).

Expression of the second key player for CP-AMPAR facilitation, polyamines, also depends on the cell type, animal age and can also be affected during pathogenesis. Although the neurons are fully equipped for polyamine synthesis and degradation, the expression of some the necessary enzymes is under strong developmental control (Bernstein and Muller, 1995). Biosynthesis of polyamines requires ornithine decarboxylase (ODC) to catalyze the formation of putrescine, which is the precursor of the other two polyamines. The biochemical and immunohistochemical studies show that in the rat brain the highest ODC activity is at P0 (Ichikawa et al., 1997). Then ODC activity rapidly declines within the first 2 postnatal weeks. In adulthood ODC immunoreactivity is strongly reduced and has region-specific and cell-type-specific expression patterns. Usage of antibodies against spermine/spermidine has confirmed different levels of polyamine synthesis in distinct neuronal populations. For instance, in the hippocampus spermine/spermidine immunolabeling of neurons located in the CA1 and CA3 regions was weaker compared to the cells in the CA2 area, and labeling of interneurons was higher relative to pyramidal cells. Similar cell-type specificity of polyamine expression was detected also in few neocortical areas, hypothalamus, nucleus ruber, dorsolateral thalamus, raphe nuclei, central and lateral amygdale, and the cerebellar cortex (Laube et al., 2002; Krauss et al., 2007).

Elevated ODC activity and putrescine concentrations have been detected in the brain in pathological conditions such as Alzheimer’s disease (AD) and ischemia (Paschen et al., 1991; Bernstein and Muller, 1995; Morrison and Kish, 1995; Keinanen et al., 1997). Nilsson et al have found evidence for translocation of ODC from the nucleus to the cytoplasm of neocortical pyramidal cells at the early stages of Alzheimer’s disease. Elevated expression of ODC was also found in cerebellar Purkinje cells and in the hippocampus (Nilsson et al., 2006). Similar alterations of ODC expression and polyamine production were observed during cerebral ischemia. The ODC mRNA levels increases after ischemia onset together with putrescine content, while spermidine and spermine levels remain constant or decline. Interestingly, in healthy tissue, increased ODC activity results in elevation of all three polyamines (Temiz et al., 2005).

Thus, both synaptic expression on CP-AMPARs and polyamine synthesis alternate during development and in response to physiological and pathophysiological stimuli. These alternations might have a significant impact on synaptic short-term plasticity at the affected synapses by means of polyamine-dependent facilitation. However, unfortunately this issue is often outside the scope of these types of studies.

### Modulation of CP-AMPAR Polyamine Sensitivity by Auxiliary Subunits

Biophysical properties of synaptic CP-AMPARs that are important for polyamine-dependent facilitation can be drastically affected by interaction with auxiliary subunits such as transmembrane AMPAR regulatory proteins (TARPs), cornichon homologues proteins (CNIHs) and germ cell specific gene 1-like protein (GSG1L). TARPs can be subdivided on the basis of functional differences and sequence homologies into the type 1 TARPs comprising the subunits γ2, γ3, γ4, and γ8, and the type II TARPs, γ5, and γ7. Type 2 TARPs modulate only CI-AMPARs (Kato et al., 2008). Among type 1 TARPs γ2 is the most studied member. Interaction of CP-AMPARs with γ2 slows desensitization and deactivation and greatly attenuates intracellular polyamine block across all voltages, most obviously at depolarized potentials (Soto et al., 2007; Soto et al., 2014). Three other members of TARP1 family, γ3, γ4, and γ8, regulate CP-AMPA receptors in a qualitatively similar manner (Cho et al., 2007). Interaction with CNIHs also reduce CP-AMPARs desensitization and polyamine sensitivity. Resent findings suggest that both TARPs and CNIHs relieve channel block by enhancing the rate of blocker permeation (Brown et al., 2018). Similarly, to type 1 TARPs and CNIHs, GSG1L slows deactivation and desensitization of CP-AMPARs (Shanks et al., 2012). However, in contrast to other auxiliary AMPAR subunits GSG1L increases intracellular polyamine block of these channels greatly suppressing outward currents in the presence of intracellular polyamines (McGee et al., 2015; Greger et al., 2017). Obviously, modulation of polyamine sensitivity and the desensitization properties of CP-AMPARs by the auxiliary subunits might have a strong influence on the rate, frequency dependence and magnitude of polyamine-dependent facilitation. These questions will have to be addressed in future studies.

## Concluding Remarks

Thus, the polyamine dependent facilitation of CP-AMPARs can play an important role as an alternative postsynaptic mechanism of short-term synaptic enhancement: (i) in a number of excitatory synapses converging to interneurons in the developing and adult brain; (ii) at connections between glutamatergic neurons during early postnatal development.

Further investigation into the role of polyamine dependent facilitation of CP-AMPARs at synapses where expressions of CP-AMPARs and polyamines are changed by network activity, homeostatically driven plasticity or neurological disorders, is essential for better understanding of the fuctioning of neuronal networks.

## Author Contributions

All authors listed have made a substantial, direct and intellectual contribution to the work, and approved it for publication.

## Conflict of Interest Statement

The authors declare that the research was conducted in the absence of any commercial or financial relationships that could be construed as a potential conflict of interest.
